# Colonization with multidrug-resistant organisms impairs survival in patients with hepatocellular carcinoma

**DOI:** 10.1007/s00432-021-03741-0

**Published:** 2021-07-20

**Authors:** Vera Himmelsbach, Mate Knabe, Phillip G. Ferstl, Kai-Henrik Peiffer, Jan A. Stratmann, Thomas A. Wichelhaus, Michael Hogardt, Volkhard A. J. Kempf, Stefan Zeuzem, Oliver Waidmann, Fabian Finkelmeier, Olivier Ballo

**Affiliations:** 1 Department of Medicine, Gastroenterology, Hepatology and Endocrinology, Goethe University Frankfurt, Frankfurt/Main, Germany; 2 Institute of Medical Microbiology and Infection Control, Goethe University Hospital, Frankfurt/Main, Germany; 3 University Center of Competence for Infection Control of the State of Hesse, Frankfurt/Main, Germany; 4 University Cancer Center, Goethe University Hospital, Frankfurt/Main, Germany; 5 Department of Medicine, Hematology/Oncology, Goethe University Hospital, Frankfurt/Main, Germany

**Keywords:** Hepatocellular carcinoma, Multidrug-resistant organisms, Infection, Antibiotic steward-ship, Antibiotics, ESBL, VRE, Survival, Liver cancer

## Abstract

**Introduction:**

MDRO-colonization has been shown to impair survival in patients with hematological malignancies and solid tumors as well as in patients with liver disease. Despite the increasing spread of multidrug-resistant organisms (MDRO), its impact on patients with hepatocellular carcinoma (HCC) has not been studied. We conducted this retrospective study to analyze the impact of MDRO-colonization on overall prognosis in HCC patients.

**Materials and methods:**

All patients with confirmed HCC diagnosed between January 2008 and December 2017 at the University Hospital Frankfurt were included in this study. HCC patients with a positive MDRO screening before or within the first 90 days after diagnosis of HCC were defined as colonized HCC patients, HCC patients with a negative MDRO screening were defined as noncolonized HCC patients.

**Results:**

59 (6%) colonized and 895 (94%) noncolonized HCC patients were included. *Enterobacterales* with extended-spectrum β-lactamase-like phenotype with or without resistance to fluoroquinolones (ESBL/ ± FQ) were the most frequently found MDRO with 59%, followed by vancomycin-resistant *Enterococcus faecium* with 37%. Colonized HCC patients had more severe cirrhosis and more advanced HCC stage compared to noncolonized HCC patients. Colonized HCC patients showed an impaired survival with a median OS of 189 days (6.3 months) compared to a median OS of 1001 days (33.4 months) in noncolonized HCC patients. MDRO-colonization was identified as an independent risk factor associated with survival in multivariate analysis.

**Conclusion:**

MDRO-colonization is an independent risk factor for survival in patients with HCC highlighting the importance of regular MDRO screening, isolation measures as well as interdisciplinary antibiotic steward-ship programs to guide responsible use of antibiotic agents.

## Introduction

Hepatocellular carcinoma (HCC) is a leading cause of cancer‐related death globally and the most common malignant primary liver cancer disease. HCC usually develops as a result of chronic liver disease due to chronic viral hepatitis, alcohol abuse and in fast increasing numbers from non-alcoholic steatohepatitis (*n*ASH). The risk of HCC development increases with the stage of liver fibrosis. The cumulative risk for HCC development in patients with established cirrhosis ranges from 5 to 30% within 5 years (Villanueva 2019). HCC treatment is performed according to the BCLC (Barcelona Clinic Liver Cancer) stages (European Association for the Study of the Liver 2018). Curative HCC treatment with local ablative procedures, surgical resection or liver transplantation is reserved for patients in early stages with sufficient liver function (European Association for the Study of the Liver 2018). For HCC patients with unresectable advanced stages palliative treatment with transarterial chemoembolization (TACE) or sorafenib has been standard of care in the past decade. In 2020 immunotherapy with the combination of atezolizumab and bevacizumab has become the favored first line treatment for these patients (Finn et al. 2020).

The global spread of multidrug-resistant organisms (MDRO), namely, vancomycin-resistant *Enterococcus faecalis/faecium* (VRE), methicillin-resistant *Staphylococcus aureus* (MRSA) and multidrug-resistant Gram-negative bacteria (MDRGN) complicates treatment and isolation measures in health care (2013). MDRO-colonization has been shown to especially impair survival in patients with hematological malignancies and solid tumors as well as in patients with liver disease (Ballo et al. 2019; Ferstl et al. 2017, 2021; Stratmann et al. 2020; Arvaniti et al. 2010; Waidmann et al. 2015). Apart from the cancer disease itself, most HCC patients also suffer from relevant chronic liver disease. Infectious complications, mostly by bacteria belong to the predominant causes of acute on chronic liver failure leading to high rates of death (Waidmann et al. 2015; Ferstl et al. 2021). As MDRO severely narrow antibiotic treatment options higher mortality rates in patients with liver disease and MDRO are reported (Waidmann et al. 2015; Ferstl et al. 2021; Fernandez et al. 2016a). Despite the knowledge of MDRO-colonization adversely affecting survival in these patients, no studies are available investigating the impact of MDRO-colonization in patients with HCC. Considering that many HCC patients do not die due to uncontrolled tumor growth but due to complications of cirrhosis (e.g., sepsis) there is a need to improve supportive measures, e.g., by avoiding MDRO-colonization and lethal infections (Couto et al. 2007).

We conducted this retrospective analysis to determine the incidence of MDRO-colonization in HCC patients and to evaluate its impact on the clinical course.

## Materials and methods

### Study design and microbiological definitions

Between January 2008 and December 2017, patients with confirmed HCC presenting at the Department of Internal Medicine 1 of the Frankfurt University Hospital were included in this study. HCC was diagnosed according to current guidelines by dynamic imaging techniques with 4‐phase multidetector computed tomography (CT) scan or dynamic contrast‐enhanced magnetic resonance imaging (MRI) and the typical hallmark of HCC (hypervascularity in the arterial phase with washout in the portal venous or delayed phases) or by histopathological examination of biopsies taken from liver tumors or metastases (European Association for the Study of the Liver 2018).

BCLC stage, model of end‐stage liver disease (MELD) score, Child–Pugh score and Albumin–Bilirubin (ALBI) grade were assessed by clinical examination, laboratory parameters and the results of ultrasound, CT scans and MRI imaging (Llovet et al. 1999; Kamath et al. 2001; Pugh et al. 1973; Johnson et al. 2015). The BCLC stage determined HCC treatment (European Association 2012). Briefly, patients with early stage HCC within the Milan criteria were either listed at Eurotransplant for liver transplantation, received resection or local ablative therapy by radiofrequency ablation (RFA). HCC patients with intermediate or advanced disease received treatment of HCC with local ablative therapy including RFA, TACE or systemic treatment as recommended by the current guidelines. Patients with end‐stage HCC received best supportive care.

The study was performed in accordance with the Declaration of Helsinki. The study was approved by the institutional review board of the Frankfurt University Hospital.

### Screening procedure and definitions

According to German infection law (Infektionsschutzgesetz, IfSG, initially decided in the year 2001) an infection control protocol to prevent the transmission of MDRO is required (Bundesministerium der Justiz und für Verbraucherschutz 2019). At the University hospital Frankfurt, this legal requirement by IfSG and the recommendations of the Commission for Hospital Hygiene and Infection Prevention (KRINKO) at the Robert Koch Institute, Berlin, Germany were updated regularly and entirely fulfilled (Robert Koch Institut 2012). Patients reporting defined risk factors, e.g., arriving from high-prevalence countries and patients, e.g., admitted to oncology wards are systematically screened for MDRO at the day of admittance by nasal, rectal and pharyngeal swabs (Reinheimer et al. 2016, 2017).

MDRO were defined as *Enterococcus faecalis* or *Enterococcus faecium* with vancomycin resistance (VRE), *Methicillin-*resistant *Staphylococcus aureus* (MRSA) and MDRGN. MDRGN were defined as *Klebsiella pneumoniae*, *Klebsiella oxytoca*, *Escherichia coli*, *Proteus mirabilis* with extended spectrum beta-lactamase (ESBL)-like phenotype as well as *Enterobacterales*, *Acinetobacter baumannii* and *Pseudomonas aeruginosa* resistant against piperacillin, any 3rd/4th generation Cephalosporin, and fluoroquinolones ± carbapenems. MDRGN with resistance against carbapenems have been described as Carbapenem-resistant *Enterobacteriaceae* (CRE) (Temkin et al. 2014).

Patients with a detection of MDRO before or within the first 90 days after diagnosis of HCC were defined as colonized HCC patients. Patients in which never a MDRO was detected were defined as noncolonized HCC patients. Patients who acquired MDRO later than 90 days after HCC diagnosis and patients that never received MDRO screening were not further investigated.

### Detection of MDRO

For MDRO, screening culture swabs were transferred from Amies collection and transport medium onto selective agar plates for the detection of VRE, MRSA and MDRGN. Species identification was performed by Matrix-assisted laser desorption ionization–time-of-flight analysis (VITEK MS, bioMérieux, Nürtingen, Germany; since the year 2011) or biochemical analysis. Antimicrobial susceptibility testing was performed according to guidelines set by Clinical and Laboratory Standards Institute (CLSI) and using VITEK 2 since the year 2010 (bioMérieux), antibiotic gradient tests or disc diffusion method.

### Statistical analysis

This study was designed as a retrospective cohort study. All patients with diagnosed HCC were retrospectively collected from the patient’s documentation system. They were followed up until death or last contact. The primary end point was overall survival. Continuous variables are shown as means ± standard deviation and categorical variables are reported as frequencies and percentages. Differences between different patient cohorts were determined using the nonparametric Wilcoxon–Mann–Whitney and Kruskal–Wallis tests. For sub‐analysis of a statistically significant Kruskal–Wallis test, the Bonferroni correction was used. *P* values < 0.05 were considered to be significant. Predictors of survival were determined using a univariate Cox regression hazard model. Death was recorded as event. For assessment of independent predictors of survival, a multivariate Cox regression hazard model with forward stepwise (likelihood ratio) entry was used. Survival curves with the estimated hazards were calculated with the Cox regression model. Statistical analyses were performed with SPSS (Version 27.0, IBM, New York, USA) and GraphPad Prism 8.0 (GraphPad Software, La Jolla, CA, USA).

## Results

### Baseline characteristics and microbiological findings

**Fig. 1:**
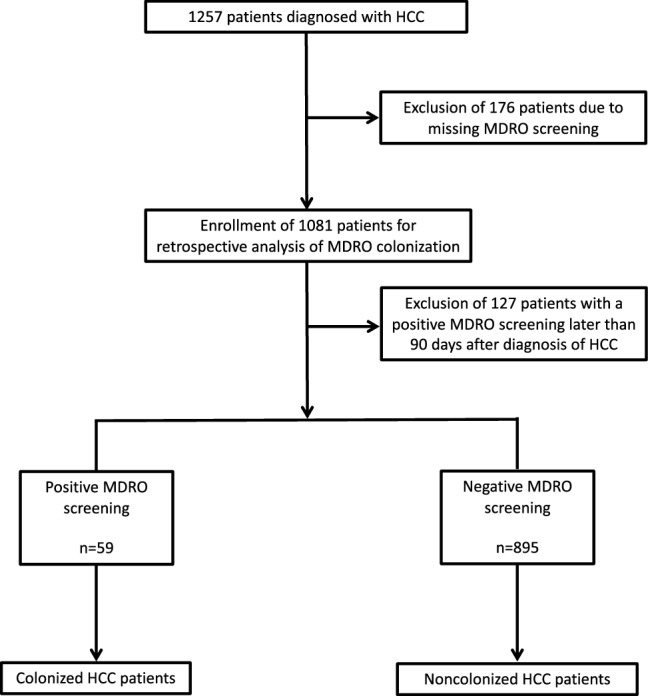
Flow sheet for screening, enrollment and allocation

1257 patients were diagnosed with HCC between 2001 and 2017 at the University Hospital Frankfurt. HCC patients that never received MDRO screening (*n* = 176) and HCC patients who acquired MDRO-colonization later than 90 days after HCC diagnosis (*n* = 127) were excluded from further analysis (Fig. 1). For the remaining 954 HCC patients the median follow-up was 418 days (range 1–6345). 59 (6%) were colonized with a MDRO and 895 (94%) were noncolonized (Table 1). Colonized as well as noncolonized HCC patients had a median age of 64 years (range 34–84 years and 20–89 years, respectively, *p* = 0.815). The most frequently found MDRO within the 59 colonized HCC patients was ESBL/ ± FQ with 35 patients (59%), 19 (54.3%) of them being *E. coli* and 16 (45.7%) being *K. pneumoniae*. The second most found MDRO was VRE with 22 patients (37%) (Table 2). MRSA colonization was found in 8 HCC patients (14%), 1 patient was colonized with a CRE (2%). 6 patients (10%) were colonized with more than 1 MDRO.

**Table 1:** Baseline characteristics

Parameter	All patients	Colonized	Noncolonized	*p* Value
Epidemiology				
Patients (*n*, %)	954	59 (6.2)	895 (93.8)	
Male sex (*n*, %)	755 (79.1)	41 (69.5)	714 (79.8)	0.069
Age (median, range)	64 (20–89)	64 (34–84)	64 (20–89)	0.815
Etiology of liver disease				
Hepatitis B (*n*, %)	177 (18.6)	9 (15.3)	168 (18.8)	0.605
Hepatitis C (*n*, %)	322 (33.8)	20 (33.9)	301 (33.6)	1.0
NASH (*n*, %)	121 (12.7)	10 (16.9)	111 (12.4)	0.421
Alcohol (*n*, %)	321 (33.7)	25 (42.4)	296 (33.1)	0.251
Cryptogenic n, %)	56 (5.9)	5 (8.5)	64 (7.2)	0.796
Other or unknown (*n*, %)	47 (4.9)	4 (6.8)	43 (4.8)	0.536
Cirrhosis (*n*, %)	875 (91.7)	56 94.9)	819 (91.5)	1.0

All *p* values reported are two-sided. Statistical significance was defined as *p* ≤ 0.05.

**Table 2:** Microbiological findings

Characteristic	Colonized
Patients (*n*, %)	59
VRE (*n*, %)	22 (37.3)
Enterococcus faecalis (*n*, %)	0 (0)
Enterococcus faecium (*n*, %)	22 (100)
ESBL/ ± FQ (*n*, %)	35 (59.3)
Escherichia coli (*n*, %)	19 (54.3)
Klebsiella pneumoniae (*n*, %)	16 (45.7)
MRSA (*n*, %)	8 (13.6)
CRE (*n*, %)	1 (1.7)
≥ 2 MDRO (*n*, %)	6 (10.2)

*VRE*, vancomycin-resistant *Enterococcus faecalis/ faecium*; *ESBL/ ± FQ*, extended-spectrum ß-lactamase phenotype with or without flourquinolone resistance; *CRE*, carbapenem-resistant Enterobacterales; *MRSA*, methicillin-resistant *S. aureus*; *MDRO*, multidrug-resistant organism

### Clinical findings

Colonized HCC patients tended to have more advanced cancer disease as well as more impaired liver function (Table 3). Namely, BCLC stage D—representing end-stage HCC disease—was found in 11 colonized HCC patients (19%), but only in 42 noncolonized HCC patients (5%) (*p* = 0.001). Analogously colonized HCC patients had more ALBI grade 3 than noncolonized HCC patients (32% vs. 9%, *p* < 0.001). Colonized HCC patients had higher Child–Pugh scores than noncolonized HCC patients (*p* < 0.001) and a higher median MELD score (14, range 6–40 vs. 10, range 6–40, *p* < 0.001). The percentage of HCC patients receiving resection (20% vs. 24%, *p* = 0.534), local ablation (19% vs. 21%, *p* = 0.743) or liver transplantation (9% vs. 10%, *p* = 1.0) was similar in both cohorts. Only 2 (3%) colonized HCC patients received systemic treatment with sorafenib compared to 88 (10%) of noncolonized HCC patients (*p* < 0.001).

**Table 3:** Clinical findings

Parameter	Colonized	Noncolonized	*p* value
Patients (*n*, %)	59 (6.2)	895 (93.8)	
BCLC stage			
0 (*n*, %)	0 (0)	2 (0.2)	0.001
A (*n*, %)	15 (25.4)	275 (30.7)	0.001
B (*n*, %)	23 (39)	390 (43.6)	0.001
C (*n*, %)	9 (15.3)	160 (17.9)	0.001
D (*n*, %)	11 (18.6)	42 (4.7)	0.001
Cirrhosis (*n*, %)	56 (94.9)	819 (91.5)	1.0
Child–Pugh score			
A (*n*, %)	19 (32.2)	469 (52.4)	< 0.001
B (*n*, %)	22 (37.3)	161 (18)	< 0.001
C (*n*, %)	11 (18.6)	22 (2.5)	< 0.001
MELD (median, range)	14 (6–40)	10 (6–40)	< 0.001
ALBI			
Grade 1 (*n*, %)	9 (15.3)	257 (28.7)	< 0.001
Grade 2 (*n*, %)	21 (35.6)	331 (37)	< 0.001
Grade 3 (*n*, %)	19 (32.2)	77 (8.6)	< 0.001
Metastasis (*n*, %)	4 (6.8)	41 (4.6)	0.353
Treatment			
Resection (*n*, %)	12 (20.3)	218 (24.4)	0.534
Local ablation (*n*, %)	11 (18.6)	189 (21.1)	0.743
Sorafenib (*n*, %)	2 (3.4)	217 (24.2)	< 0.001
Liver transplantation (*n*, %)	5 (8.5)	88 (9.8)	1.0

All *p* values reported are two-sided

Statistical significance was defined as *p* ≤ 0.05

### Outcome

**Fig. 2:**
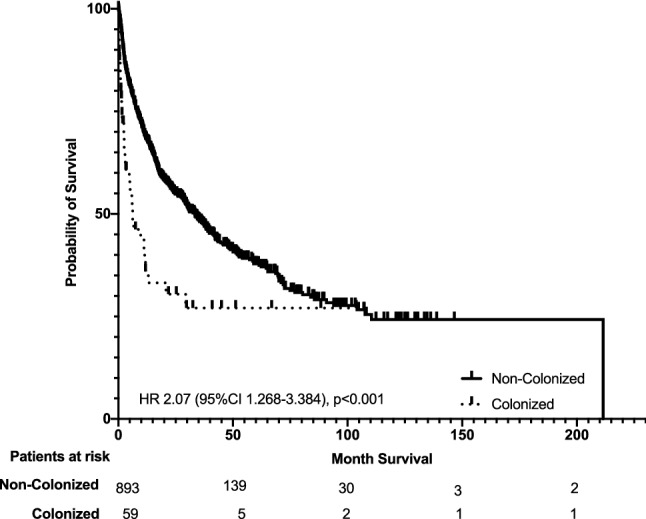
Kaplan–Meier curves for overall survival (OS). OS of colonized (dotted line) and noncolonized (solid line) HCC patients

We hypothesized that MDRO-colonization might be of prognostic value in HCC patients. Therefore, the overall survival (OS) rates of colonized and noncolonized HCC patients were compared. Colonized HCC patients had a lower median OS of 189 days (6.3 months) (95% confidence interval (CI) 18–360 days) compared to a median OS of 1001 days (33.4 months) (95% CI 812–1190 days) in noncolonized HCC patients (Fig. 2). Overall, 476 HCC patients died (33 colonized and 443 noncolonized HCC patients). Regarding the causes of death in the 33 colonized HCC patients, we found 8 (24.2%) death events to be linked to infectious complications. 6 (18.2%) patients died in a setting of uncontrolled tumor disease. 4 (12.1%) HCC patients had lethal bleeding events and 3 (9.1%) patients died from acute on chronic liver failure. In 12 (38.7%) patients appropriately determining the cause of death was not possible.

To further analyze MDRO-colonization as a prognostic parameter in HCC patients a multivariate Cox regression model with forward stepwise likelihood ratio was performed. The nominal dichotome variables age above 65 years, male sex, underlying cirrhosis, Child–Pugh class C vs. A or B, BCLC stage C or D vs. A or B, ALBI grade 3 vs. grade 1 or 2, alpha-fetoprotein (AFP) > 400 ng/ml, sorafenib treatment, resection as HCC treatment and MDRO-colonization were included in this model. As shown in Table 4, in addition to the well-known risk factors in HCC, such as an advanced BCLC stage, an ALBI grade 3 or an AFP > 400 ng/ml, the colonization with an MDRO was an independent risk factor associated with a worsened overall survival.

**Table 4:** Univariate and multivariate analyses associated with survival in HCC patients

Parameter	HR	95% CI	*P* value	HR	95% CI	*P* value
	Univariate analysis			Multivariate analysis		
Age > 65 years	1.062	0.886–1.062	0.515			
Male sex	1.146	0.917–1.433	0.229			
Underlying cirrhosis	1.383	0.917–2.086	0.122			
Child–Pugh class C vs. A or B	1.216	0.902–1.640	0.200			
BCLC stage C or D vs. A or B	3.193	2.608–3.909	< 0.001	2.426	1.888–3.118	< 0.001
ALBI grade 3 vs. grade 1 or 2	2.972	2.254–3.919	< 0.001	1.871	1.363–2.570	< 0.001
Alpha-fetoprotein > 400 ng/ml	2.605	2.112–3.214	< 0.001	2.336	1.841–2.965	< 0.001
Sorafenib treatment	0.905	0.825–1.242	0.905			
Resection as HCC treatment	0.489	0.389–0.614	< 0.001	0.553	0.415–0.737	< 0.001
MDRO-colonization	2.077	1.457–2.962	< 0.001	1.653	1.089–2.509	0.018

All *p* values reported are two-sided

Statistical significance was defined as *p* ≤ 0.05

*CI* confidence interval, *HR* hazard ratio

## Discussion

Several studies have shown that MDRO-colonization adversely affects the clinical course of patients with hematological malignancies and solid tumors as well as in patients with liver disease (Ballo et al. 2019; Ferstl et al. 2017; Stratmann et al. 2020; Arvaniti et al. 2010). Although HCC is a leading cause of cancer‐related death globally, to our knowledge this is the first study investigating the clinical impact of MDRO-colonization on patients with HCC.

Due to strict exclusion criteria (exclusion of HCC patients that never received MDRO screening and HCC patients who acquired MDRO-colonization later than 90 days after HCC diagnosis) we were able to define two clearly separated HCC patient cohorts (Fig. 1). By excluding HCC patients that were never screened for MDRO-colonization the risk of false-negative or occult positive noncolonized HCC patients was minimized. Excluded HCC patients that acquired MDRO-colonization later than 90 days after diagnosis of HCC prevented bias and confounders, as in these patients the clinical course is not primarily or at least not in the beginning determined by MDRO-colonization.

After exclusion of the above patients we found 59 (6%) of 954 HCC patients to be MDRO-colonized and 895 (94%) HCC patients to be noncolonized. The most frequent MDRO detected in colonized HCC patients was ESBL/ ± FQ with 59%, followed by VRE with 37%. The finding of ESBL as the most frequent MDRO in HCC patients is not surprising, since other studies analyzing the epidemiology of MDRO in Europe already described ESBL as a frequently isolated MDRO in hepatological patients (Fernandez et al. 2016b; Merli et al. 2015). A dominant prevalence of VRE-colonization was also expected. Germany has one of the highest VRE prevalence in Europe and again Hesse, North Rhine-Westphalia, Thuringia and Saxony have the highest proportion of VRE-colonization within Germany (Gastmeier et al. 2014). Surprisingly, colonization with CRE—known to cause untreatable lethal infections in patients with liver and other diseases—was only found in one HCC patients (Ballo et al. 2019; Ferstl et al. 2017).

Colonized patients with HCC had strongly impaired prognosis with a median OS of 189 days (6.3 months) compared to a median OS of 1001 days (33.4 months) in noncolonized HCC patients. The vast difference in survival for colonized and noncolonized HCC patients is due to different reasons. As shown in Table 3 cirrhosis and cancer disease were significantly more advanced in colonized HCC patients. Patients suffering from more severe cirrhosis and/or more severe HCC do often not qualify for systemic treatment and have impaired survival regardless of MDRO-colonization. They are more likely to receive antibiotic treatment, they are more often hospitalized and treated on intensive care unit and in turn more often provided with invasive devices (e.g., urinary catheter, central venous catheter etc.). All these factors impair survival and are likewise known to be risk factors for MDRO-colonization (Buul et al. 2012). Therefore, highest importance is attached to the multivariate analysis of factors associated with survival in this study. All relevant characteristics collected in our large HCC data base that potentially affect survival were included. Here, besides BCLC stage C or D, ALBI grade 3, AFP > 400 ng/ml and resection as HCC treatment, MDRO-colonization was identified as an independent prognostic factor in HCC patients. As causes for death in HCC patients vary amongst infectious complications, liver or multiple organ failure (Arroyo et al. 2020), uncontrolled tumor growth, lethal bleeding events and more (each of them possibly leading to one another), unambiguously determining the leading cause for death in HCC patients is difficult in a retrospective study setting and needs to be addressed in future prospective studies. We found, however, that only 6 (18.2%) HCC patients died in a setting of uncontrolled tumor disease. In at least 8 (24.2%) colonized HCC patients, death events were associated with infectious complications. Couto et al. showed that up to 43% of HCC patients do not die as a result of cancer progression, but from complications of the underlying cirrhosis (sepsis, bleeding etc.) (Couto et al. 2007). The fact that many HCC patients die in the absence of uncontrolled tumor growth emphasizes the importance of our finding in clinical practice even more.

At our hospital it was already reported that MDRO-colonization adversely affects survival in patients with hematological malignancies and solid tumors as well as in patients with liver disease (Ballo et al. 2019; Ferstl et al. 2017; Stratmann et al. 2020; Arvaniti et al. 2010). This is the first study proving a similar impact of MDRO-colonization on patients with HCC. Hospital antibiotic stewardship programs have shown to reduce colonization and infection with MDRO (Baur et al. 2017). Thus, clinicians should carefully balance the risks and benefits of intensive antibiotic agents to reduce incidence of MDRO colonization and infection. In summary, this study highlights the importance of MDRO screening, appropriate isolation measures as well as interdisciplinary antibiotic steward-ship programs in the context of HCC treatment, as these patients are highly susceptible to infections due to cancer and liver disease.

## Data Availability

The data that support the findings of this study are available from the corresponding author upon reasonable request.

## Abbreviations

AFP: Alpha-fetoprotein
ALBI: Albumin–Bilirubin
BCLC: Barcelona clinic liver cancer
CI: Confidence interval
CPS: Child–Pugh stage
CR: Complete response
CRE: Carbapenem-resistant *Enterobacterales*
CT: Computed tomography
ESBL/ ± FQ: *Enterobacterales* With extended-spectrum β-lactamase-like phenotype with or without resistance to fluoroquinolones
HCC: Hepatocellular carcinoma
MDRO: Multidrug-resistant organisms
MRI: Magnetic resonance imaging
MRSA: Methicillin-resistant *Staphylococcus aureus*
MDRGN: Multidrug-resistant Gram-negative bacteria
MELD: Model of end‐stage liver disease
NASH: Non-alcoholic steatohepatitis
OS: Overall survival
TACE: Transarterial chemoembolization
VRE: Vancomycin-resistant *Enterococcus faecalis/faecium*
